# Simple, rapid and efficient transformation of genotype Nisqually-1: a basic tool for the first sequenced model tree

**DOI:** 10.1038/s41598-017-02651-x

**Published:** 2017-06-01

**Authors:** Shujuan Li, Cheng Zhen, Wenjing Xu, Chong Wang, Yuxiang Cheng

**Affiliations:** State Key Laboratory of Tree Genetics and Breeding, Northeast Forestry University, 26 Hexing Road, Harbin, 150040 China

## Abstract

Genotype Nisqually-1 is the first model woody plant with an available well-annotated genome. Nevertheless, a simple and rapid transformation of Nisqually-1 remains to be established. Here, we developed a novel shoot regeneration method for Nisqually-1 using leaf petiole and stem segment explants. Numerous shoots formed in the incision of explants within two weeks. The optimized shoot regeneration medium (SRM) contained 0.03 mg l^−1^ 6-benzylaminopurine, 0.02 mg l^−1^ indole-3-butyric acid and 0.0008 mg l^−1^ thidiazuron. Based on this, *Agrobacterium*-mediated genetic transformation of stem explants was examined using the vector pBI_121_ that contains the β-glucuronidase (GUS) as a reporter gene. Consequently, factors affecting transformation frequency of GUS-positive shoots were optimized as follows: *Agrobacteria* cell suspension with an OD_600_ of 0.4, 20 min infection time, 2 days of co-cultivation duration and the addition of 80 µM acetosyringone into *Agrobacteria* infective suspension and co-cultivation SRM. Using this optimized method, transgenic plantlets of Nisqually-1 – with an average transformation frequency of 26.7% – were obtained with 2 months. Southern blot and GUS activity staining confirmed the integration of the foreign *GUS* gene into Nisqually-1. This novel transformation system for Nisqually-1 was rapid, efficient, and simple to operate and will improve more genetic applications in this model tree.

## Introduction

Forests provide humanity with many benefits including clean air, lumber, and fuels, etc. Forest trees evolve many specific traits such as large wood formation, perennial growth and adaptability of seasonality^1^. Many of these features distinguish the trees from herbaceous plants and present challenges to the study of the genetic, cellular and molecular mechanisms that underlie the unique tree biology^2, 3^. However, several factors such as few model tree species, less availability of full-genome sequences of trees and a deficiency of genetic tools for trees, significantly limit the biological improvement of individual trees.

Nisqually-1, a female clone of black cottonwood (*Populus trichocarpa*), was selected as the first tree species for genome sequencing and its complete genome was reported in ref. 4. Since the release of its genome sequence, *P. trichocarpa* has served as a model tree species for a variety of studies on the unique growth and developmental traits, molecular physiologies and ecological functions of the trees^5, 6^. Additionally, many functional genomic studies, especially in numerous gene families, have been reported in *P. trichocarpa* ^7–9^. Nevertheless, there is very little literature on molecular mechanisms of the functional genes in this model tree and the genetic functions (loss-of-function and gain-of-function) reported have mostly been investigated in *Populus* hybrids, not in Nisqually-1. The lack of studies in Nisqually-1 could be attributed to difficult or time-consuming genetic transformation for Nisqually-1 unlike many *Populus* hybrids. Such recalcitrance of this model tree has been an impediment to the utilization of its well-annotated genomic resource.

To date, two methods have been reported on genetic transformation of Nisqually-1. The one method from Ma *et al*.^10^ includes two processes: inducing transgenic calli and regenerating shoots. In this method, transgenic calli were induced from stem segment explants by naphthaleneacetic acid (NAA) and 2iP plant growth regulators (PGRs) and then shoots were regenerated from transgenic calli undergoing two types of MS media supplemented with different concentration of thidiazuron (TDZ). The other method as described by Song *et al*.^11^ and Li *et al*.^12^ also involves transgenic calli induction and shoot regeneration. Transgenic calli were induced by kinetin and 2,4-dichlorophenoxyacetic acid (2,4-D) from stem segments of the 5^th^ to 8^th^ internodes harvested from 5- to 6-month-old greenhouse-grown young trees of Nisqually-1. Overall, the two successful methods provide approximately 6% and 12% transformation efficiencies of Nisqually-1 over a relatively long period of time (5 to 6 months). To improve time efficiency, a short-period transformation method simple to operate would be valuable for facilitating molecular and genetic studies on the unique traits of this first model tree.

The objective of this study was to develop a simple, rapid and efficient genetic transformation for Nisqually-1. We first established a novel efficient shoot regeneration method for Nisqually-1 where multiple shoots were directly regenerated from each incision of leaf petiole and stem segment explants within 2 weeks. Based on this, we developed an *Agrobacterium*-mediated transformation method for Nisqually-1. The efficiency of genetic transformation was approximately 27% and the transgenic plantlets were produced with 2 months. This simple, rapid and efficient transformation of Nisqually-1 significantly facilitates gene functional studies in this first-sequenced model tree.

## Results

### Establishment of a rapid and efficient shoot regeneration protocol

The objective of this study was to establish shoot regeneration with a one-step method to facilitate the rapid transformation of Nisqually-1. Five groups of PGR combinations among TDZ, 6-BA, NAA and IBA were tested for their effects on shoot regeneration (Supplementary Table S1). The combinations of one type of cytokinin and one type of auxin (TDZ/NAA, 6-BA/NAA or 6-BA/IBA) resulted in no shoot or few shoots regenerated from the stem explants. We further tested the combinations of two types of cytokinin and one type of auxin on shoot regeneration. The results showed that a combination of 6-BA, IBA and TDZ significantly promoted multiple shoot organogenesis from stem explants of Nisqually-1.

**Figure 1:**
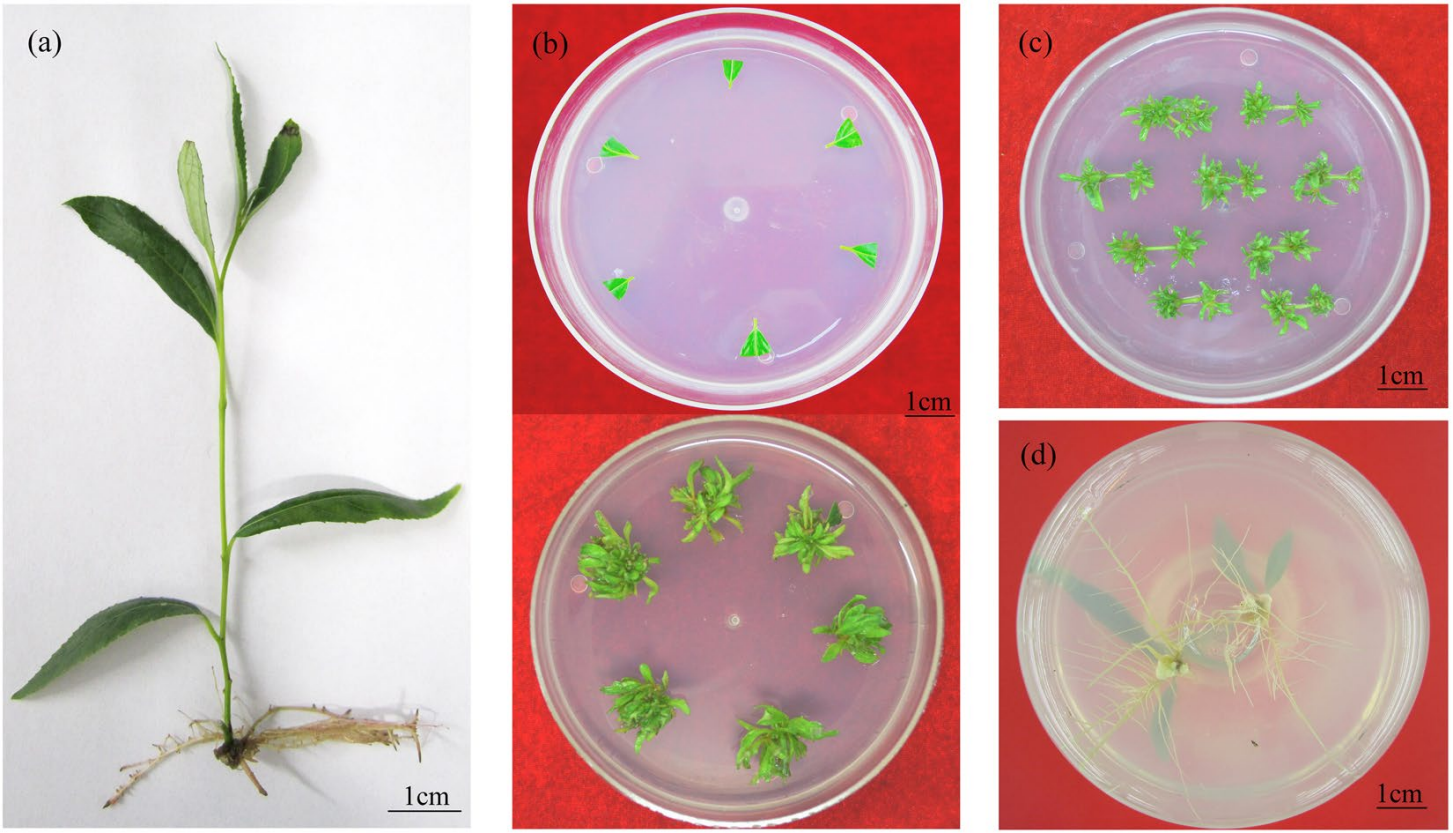
High-frequency plant regeneration of Nisqually-1. (**a**) Individual sterile 4-week-old plantlet with 5 internodes and 6 leaves used for shoot regeneration. (**b**,**c**) Multiple shoots induced from leaf petiole and internode stem segment explants, respectively. (**d**) The rooting of the regenerated shoots.

Based on the above experiments, the 6-BA, IBA and TDZ concentrations for shoot regeneration were focused on a small scale. Combined with 6-BA set to 0.03 mg l^−1^, the effects of different concentrations of IBA and TDZ on shoot regeneration from stem segment explants were evaluated (Table 1). Only two cytokinins, 6-BA and TDZ, could induce adventitious shoots from the explants while no shoot were observed in the absence of TDZ. Very low levels of TDZ in SRM greatly improved the frequency of shoot regeneration and the number of shoots. For example, in the presence of 0.0008 mg l^−1^ TDZ, the frequency reached 100% and 13 shoots per explant were regenerated on an average. However, these shoots emerged with a severe vitrification appearance. Further, our data showed that auxin IBA was crucial for shoot generation and healthy shoots. Consequently, regeneration frequency, shoot number and shoot status were optimal when the SRM included 0.03 mg l^−1^ 6-BA, 0.02 mg l^−1^ IBA and 0.0008 mg l^−1^ TDZ. On optimal SRM, the incision of stem explants expanded after 4–5 days; numerous shoots formed in the incision within 14 days; and the average number and length of shoots were 16.67 and 1.15 cm, respectively, within 25 days (Table 1; Fig. 1c). Likewise, using the leaves as explants, a number of healthy shoots formed in the petiole end of each leaf on optimal SRM containing 0.04 mg l^−1^ 6-BA, 0.02 mg l^−1^ IBA and 0.0008 mg l^−1^ TDZ (Fig. 1b). The shoots regenerated from explants were divided and transferred to RM. The roots appeared within a week and the rooting rate was 100% (Fig. 1d).

**Table 1:** Effect of 6-BA, IBA and TDZ on shoot regeneration from the stem segments of Nisqually-1 within 25 days of culture.

6-BA (mg l^−1^)	IBA (mg l^−1^)	TDZ (mg l^−1^)	Regeneration frequency (%)	Shoot number	Shoot length (cm)	Shoot status
0.03	0	0	0.00 ± 0.00j	0.00 ± 0.00g	0.00 ± 0.00h	—
0.03	0	0.0004	87.33 ± 2.31c	7.93 ± 0.25f	0.64 ± 0.009f	vitrified
0.03	0	0.0008	100.00 ± 0.00a	13.58 ± 0.25c	0.96 ± 0.04d	vitrified
0.03	0	0.0015	92.00 ± 2.00b	5.13 ± 0.55h	0.42 ± 0.035j	few, short
0.03	0.01	0	8.67 ± 1.15 f	0.50 ± 0.10ig	1.09 ± 0.06bc	seldom
0.03	0.01	0.0004	93.3 ± 3.06b	9.33 ± 0.66e	0.80 ± 0.03e	few
0.03	0.01	0.0008	100.00 ± 0.00a	15.77 ± 0.40b	1.03 ± 0.07cd	vitrified
0.03	0.01	0.0015	98.00 ± 3.46a	5.20 ± 0.46h	0.48 ± 0.07j	few, short
0.03	0.02	0	17.33 ± 1.15e	0.93 ± 0.16i	1.23 ± 0.046a	seldom
0.03	0.02	0.0004	100.00 ± 0.00a	11.00 ± 0.89d	0.82 ± 0.060e	few
0.03	0.02	0.0008	100.00 ± 0.00a	16.67 ± 0.35a	1.15 ± 0.040b	multiple
0.03	0.02	0.0015	94.67 ± 1.15b	5.20 ± 0.36h	0.61 ± 0.030f	few, short
0.03	0.04	0	24.00 ± 2.00d	1.10 ± 0.17i	1.27 ± 0.076a	seldom
0.03	0.04	0.0004	100.00 ± 0.00a	10.33 ± 0.85d	0.79 ± 0.021e	few
0.03	0.04	0.0008	100.00 ± 0.00a	16.40 ± 0.52ab	1.07 ± 0.023bc	vitrified
0.03	0.04	0.0015	100.00 ± 0.00a	6.27 ± 0.87j	0.57 ± 0.065f	few, short

Values are means ± standard error of three replicates. Each hormone treatment contained forty-five explants. Values with the different letters indicate statistical significance at *P* < 0.05 level (Duncan’s multiple range test).

Furthermore, the regenerating potential of different stem internodes from apical bud to the base of each individual plantlet was examined. The data demonstrated that many healthy shoots were regenerated from the 2^nd^ and 3^rd^ internode stem segments while the other internode stem segments showed a low potential of shoot regeneration (Supplementary Table S2). Similarly, the petioles of the 3^rd^, 4^th^ and 5^th^ leaves exhibited more potential for shoot regeneration than other leaves (Supplementary Table S3). These results suggest that moderate lignifying stem segments (or leaf petioles) possess more potential for shoot regeneration than juvenile or excessively lignified stem segments (or leaf petioles). Therefore, 2^nd^ and 3^rd^ internode stem segments or the petioles of the 3^rd^, 4^th^ and 5^th^ leaves were used as optimal explants for transformation selection and *Agrobacterium*-mediated transformation experiments.

### Determination of kanamycin concentration for transformation selection

Stem segment explants were inoculated into optimal SRM supplemented with different concentrations of kanamycin. The result showed that shoot regeneration from the explants was sensitive to kanamycin (Supplementary Figure S1). Only a few abnormal shoots were induced when the optimal SRM contained 10 mg l^−1^ kanamycin. Shoot regeneration from the explants was completely inhibited by 30 mg l^−1^ kanamycin. Meanwhile, the explants became brown within 4 weeks. Hence, 30 mg l^−1^ kanamycin was determined to select transgenic shoot generation. For rooting selection on RM, a 20 mg l^−1^ kanamycin concentration resulted in no rooting of all shoots tested (Supplementary Figure S2), which is suitable for rooting of transgenic plants because it completely prevented non-transformed shoots from rooting. Moreover, shoot regeneration was not significantly inhibited by 100, 250, 400 or 500 mg l^−1^ cefotaxime (data not shown). Given that 250 mg l^−1^ cefotaxime effectively inhibited the growth of *Agrobacterium*, it was supplemented in optimal SRM during genetic transformation selection.

### Optimization of the transformation procedure

**Figure 2:**
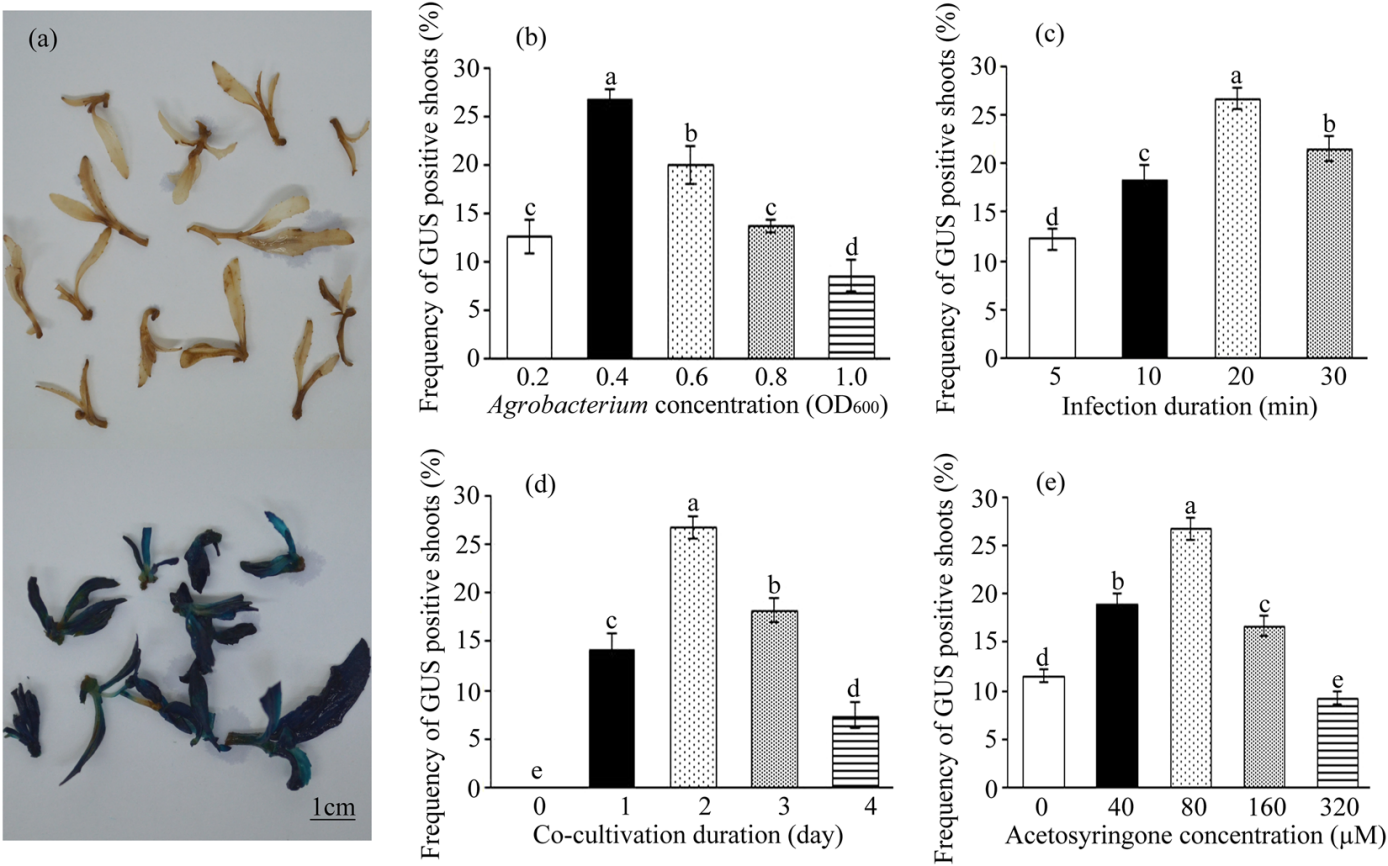
Factors that affect the frequency of GUS-positive kanamycin-resistant shoots in Nisqually-1. (**a**) GUS staining in wild-type (in top) and transgenic plants (in the bottom). (**b**–**e**) *Agrobacterium* concentration, infection duration, co-cultivation duration and acetosyringone concentration, respectively. The results are presented as the means and standard errors from three independent experiments. Within each variable, values with the different letters indicate statistical significance at *P* < 0.05 level (Duncan’s multiple range test).

An optimal SRM for stem explants was adopted to test *Agrobacterium*-mediated genetic transformation of Nisqually-1. Using CaMV 35S::GUS binary vector (Supplementary Figure S3), several factors affecting transformation frequency were optimized including *Agrobacteria* concentration, infection time, co-cultivation duration and AS concentration. Analysis of GUS staining revealed GUS expression in kanamycin-resistant shoots, but not in control shoots (Fig. 2a). For *Agrobacteria* concentration, stem segments infected by an OD_600_ of 0.4 resulted in the highest efficiency in producing transgenic shoots (Fig. 2b). For infection time, 20 min was the most suitable for infecting stem segments with the 0.4 OD_600_ *Agrobacteria* concentration (Fig. 2c). Co-cultivation duration obviously affected transformation efficiency and 2 days of co-cultivation resulted in the highest frequency of GUS-positive shoots (Fig. 2d). Moreover, the addition of AS into *Agrobacterium* suspension and co-cultivation SRM greatly improved the transformation frequency. The frequency peaked when the concentration of AS was 80 µM (Fig. 2e).

### Production and confirmation of transgenic plants

**Figure 3:**
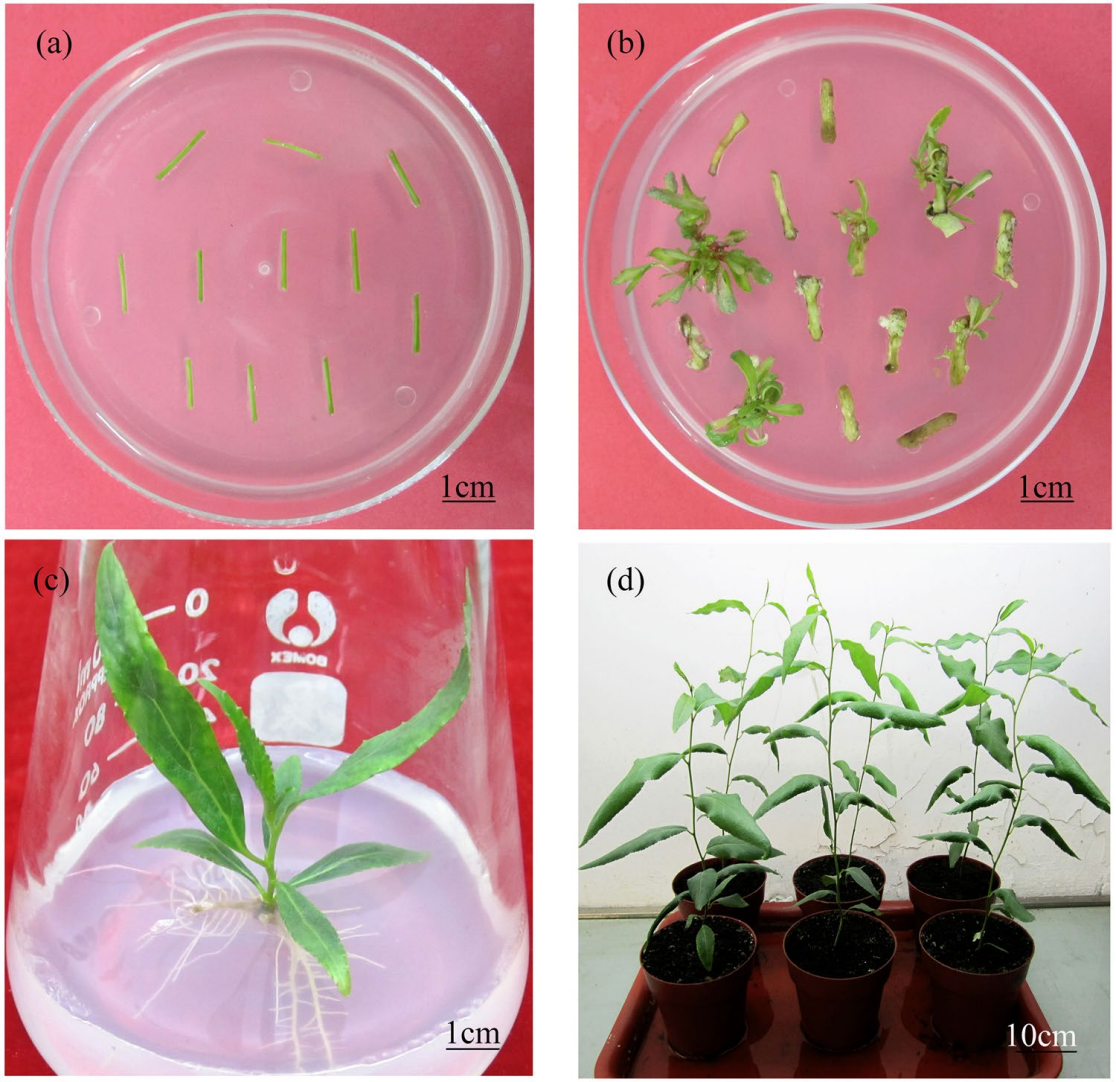
Regeneration of transgenic Nisqually-1 plants using *Agrobacterium*-mediated transformation system. (**a**) Internode stem segment explants infected were co-cultivated for 2 days. (**b**) Internode stem segment explants were cultivated on the selection medium for 40 days. (**c**) Transgenic shoots were transferred to the rooting medium with kanamycin and cefotaxime and cultured for 20 days. (**d**) Two-month-old transgenic plants grown on soil in a greenhouse.

Based on the optimized transformation system of Nisqually-1, 105 stem explants were infected with *Agrobacterium* carrying CaMV 35S::GUS binary vector. Within ~40 days, 29 independent kanamycin-resistant shoots were regenerated on the selection medium (Fig. 3a,b). Afterwards, 28 shoots rooted normally on RM containing kanamycin within 3 weeks (Fig. 3c). Nevertheless, one shoot did not root and was chlorotic, suggesting that it might be a chimera. When these 28 transgenic plantlets reached a height of 6–8 cm on RM, they were transplanted in the greenhouse, grown on soil for 8 weeks (Fig. 3d) and used for molecular confirmation of the transgene.

**Figure 4:**
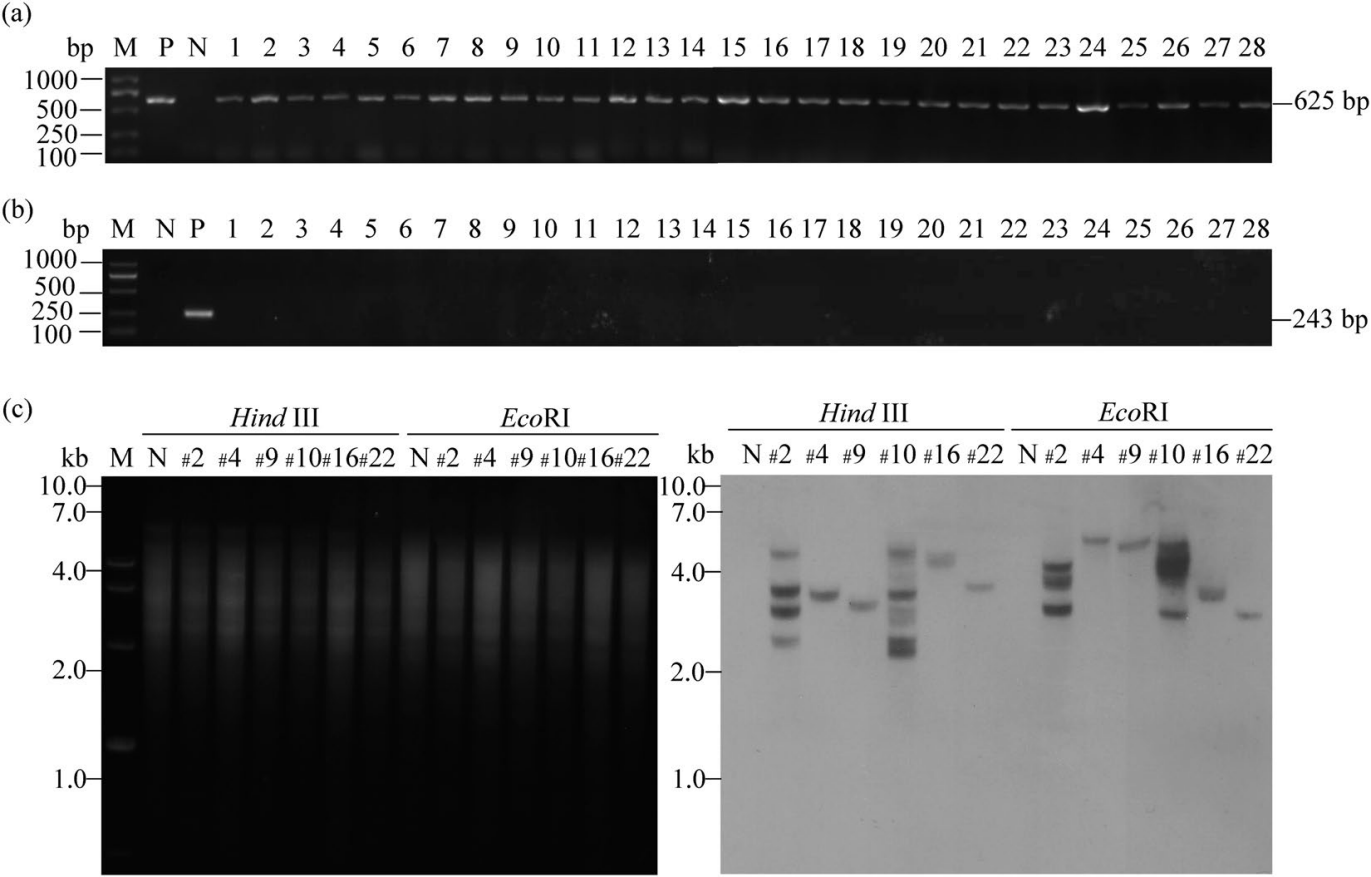
Molecular analyses of CaMV 35S::GUS transgenic Nisqually-1 plants. (**a**,**b**) PCR amplification of the *GUS* (625 bp) and *npt* III genes (243 bp) in all the 28 transgenic lines using genomic DNAs as templates. M, DNA marker; P, CaMV 35S::GUS binary vector; N, non-transformed plants; 1 to 28, all the independent transgenic lines. (**c**) Southern blot analysis of 6 transgenic lines randomly chosen. Genomic DNA was digested with *Eco*RI or *Hind* III (in the left) and probed with a DIG-labeled 625-bp GUS DNA probe (in the right). M, DNA marker; N, non-transformed plants; #2, 4, 9, 10, 16, 22, six transgenic lines. The number of bands reflects the number of transgene copies.

**Figure 5:**
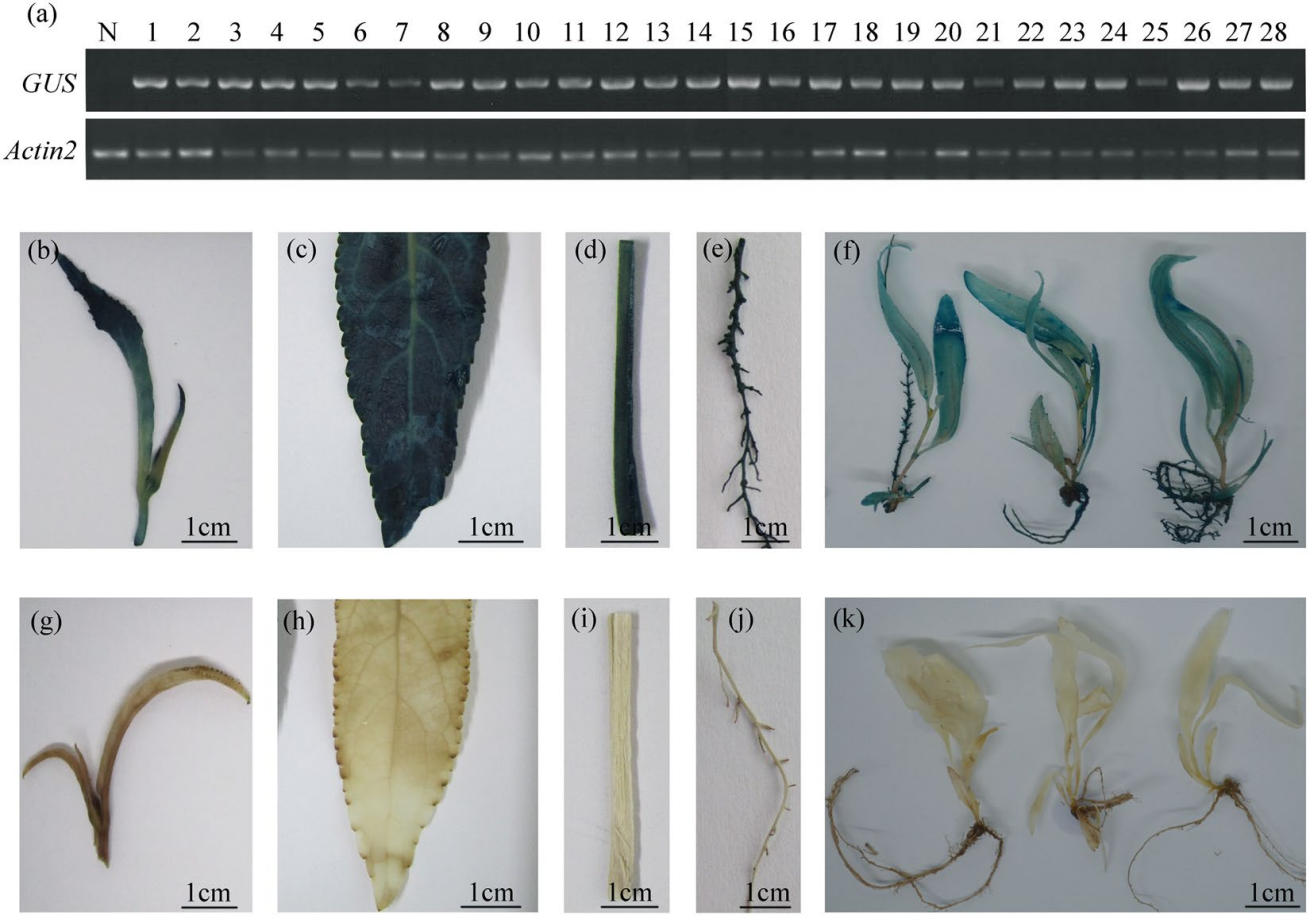
GUS expression in transgenic Nisqually-1 plants. (**a**) Analysis of *GUS* transcriptional levels in non-transformed plants (N) and transgenic lines (1 to 28) using RT-PCR analysis. The expression of *Actin2* was used as an internal control. (**b**–**k**) Histochemical GUS assay in transgenic and non-transgenic plants. GUS staining was observed in the apical bud, leaf, stem and root tissues of 8-week-old transgenic plants on soil (**b**–**e**) and 4-week-old transgenic plantlets in the rooting medium (**f**), but not observed in non-transgenic plants (**g**–**j**,**k**).

To confirm the insertion of the *GUS* gene into the genome of Nisqually-1, the transgene was examined using *GUS*-specific primers by genomic DNA PCR analysis. A 625 bp band was detected in all 28 transgenic lines on soil and not in wild-type plants, indicating the 100% frequency of PCR-positive transformants (Fig. 4a). The *npt* III gene of the pBI_121_ binary vector does not locate between LB and RB regions; therefore, it cannot be transformed into the genome of transgenic lines. Consequently, a 243 bp band of *npt* III was not detected in these transformants (Fig. 4b), excluding the possibility that it resulted from contamination of *Agrobacteria* cells that might be alive in transformants. For Southern blot analysis, hybridization signal was detected in all of the 6 independent transgenic young trees tested and the number of transgene copies varied with one to four (Fig. 4c). Next, expression of the *GUS* gene was analyzed in these young transgenic trees. Our data revealed the *GUS* expression in all 28 transgenic young trees at the transcriptional level (Fig. 5a). Furthermore, strong GUS staining signal was detected in various tissues of young transgenic trees at the protein expression level (Fig. 5b–k). These results demonstrated that the *GUS* as a foreign gene was successfully integrated into the genome of Nisqually-1 and normally expressed in young transgenic trees tested.

## Discussion

**Figure 6:**
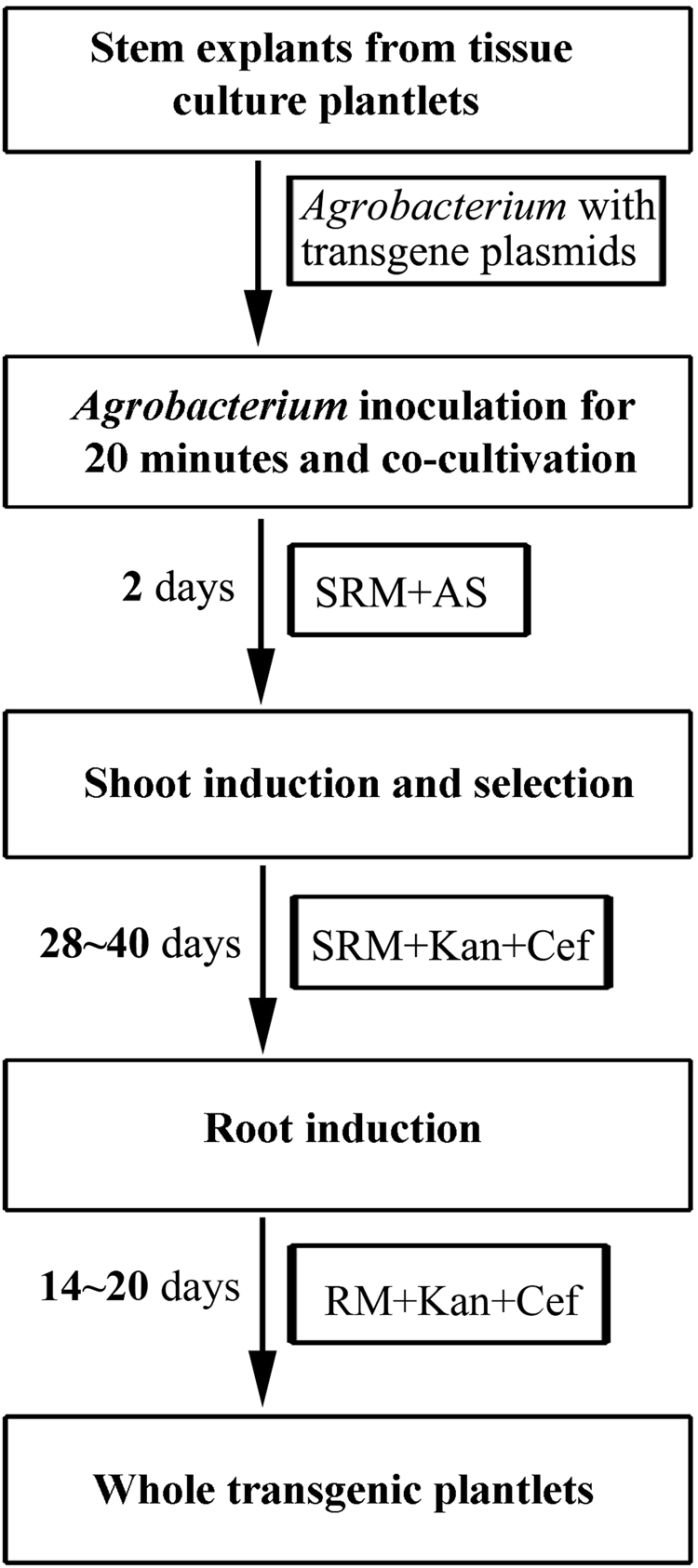
Stepwise protocol for transforming Nisqually-1 using internode stem segments as the explants. RM, rooting medium; SRM, shoot regeneration medium; AS, acetosyringone; Kan, kanamycin; Cef, cefotaxime.

Several advances have been made in tree biology research since the complete genome of clone Nisqually-1, the first model tree species, was published in ref. 4. Nevertheless, the development of more practicable genetic tools in this model tree would accelerate research studies in tree biology. For example, a simple and rapid transformation of *Arabidopsis* using in planta floral dip was in common use^13^, which has significantly contributed to the information obtained from this herbaceous model plant. Although some *Populus* species and their hybrids have been transformed successfully^14–18^, the recalcitrance of Nisqually-1 to transform is an impediment in the utilization of its genomic resources. In this study, we developed a novel *Agrobacterium*-mediated transformation system for Nisqually-1, which embodies three merits (Fig. 6). First, it shortened the time to produce Nisqually-1 transgenic plants with approximately 2 months, thus, saving time for studying tree biology at the molecular level. Second, transgenic shoots were induced in one-step from the inoculated explants, demonstrating the ease and simplicity of the system for reproducibility. Finally, it provides 26.7% transformation frequency on an average, exhibiting high efficiency in the transgene of Nisqually-1.

A highly-efficient plant regeneration method is the first step to develop its successful genetic transformation system. We developed a novel method for rapid regeneration of Nisqually-1 and numerous shoots formed in the stem segment or leaf petiole explants within 25 days (Fig. 1), which is crucial for shortening its transgene period. Optimal SRM for shoot regeneration depends on the various combinations of three PGRs namely 6-BA, IBA and TDZ. Regeneration of shoots from Nisqually-1 explants was significantly promoted by 6-BA and TDZ mixture (Table 1). TDZ has been reported as an efficient cytokinin that induces shoot regeneration in some plant species^19–23^. The concentration of TDZ for shoot regeneration in these plant species reaches to ~μM grade. However, optimal TDZ concentration for regeneration of Nisqually-1 was extremely low (0.0008 mg l^−1^, equal to 3.6 nM) in this study, which might be one of the key factors for *in vitro* shoot organogenesis. Without PGR auxin, the mix of cytokinins TDZ and 6-BA induced significantly vitrified shoots (Table 1). A combination of 0.02 mg l^−1^ IBA in SRM with cytokinins TDZ and 6-BA generated healthy shoots. Curiously, NAA did not completely substitute for the role of IBA in shoot organogenesis of Nisqually-1. Moreover, the 2^nd^ and 3^rd^ internode stem segments (or the petioles of the 3^rd^, 4^th^ and 5^th^ leaves) were identified as optimal explants for shoot regeneration, suggesting that the developmental phase of explants is a crucial factor for efficient regeneration.

Several factors affecting transformation frequency of Nisqually-1 were evaluated in the present study. The *Agrobacterium* strain is an important factor affecting the transformation efficiency in *Populus* genus^24–26^. *Agrobacterium* strains C58 or GV3101 efficiently transform Nisqually-1^10, 12^; thus, we used GV3101 in this study. Different optical densities of *Agrobacterium* suspensions during infection of explants affect plant transformation efficiency. The highest frequency was observed when an OD_600_ of 0.4 *Agrobacteria* was used (Fig. 2b). Ma *et al*.^10^ infected explants with a density of 0.5–0.6 *Agrobacterium* suspension, while Li *et al*.^12^ directly used *Agrobacterium* cultures (~0.4 OD_600_) for infecting explants. In other *populus* species or hybrids, optimal optical densities of *Agrobacterium* suspensions changed from 0.3 to 1.0 OD_600_^14, 18, 25, 27–29^. Both infection duration and co-culture duration, influence *Agrobacterium*-plant cell interactions, altering transformation efficiency. In this study, infecting explants for 20 min with *Agrobacteria* (0.4 OD_600_) resulted in the highest frequency (Fig. 2c). In comparison with previous studies^25, 30, 31^, we suggest that different poplar species benefit from different durations of *Agrobacterium* infection. Additionally, the highest transformation frequency resulted from 2 days of co-cultivation (Fig. 2d), which is in agreement with the results of Ma *et al*.^10^ and Li *et al*.^12^. The addition of AS into the *Agrobacterium* suspension and co-cultivation significantly improved transformation frequency of Nisqually-1 (Fig. 2e). Plant genetic transformation depends on the activation of the *vir* gene, which is triggered by phenols, while AS as a phenol can induce *vir* gene expression^32–34^.

Direct organogenesis is more beneficial for transgenes from trees due to its time-saving trait and the probability of lower somaclonal variations. However, some studies have reported that the high percentage of non-transgenic trees is because of their bypassing antibiotic selection through direct organogenesis^18, 35^. Of all the 29 shoots regenerated in this study, one non-transgenic shoot escaped from the selection medium, suggesting that a low percentage of non-transgenic plants occurred in the transformation of Nisqually-1. Nevertheless, this escaped non-transgenic shoot did not root on RM with 20 mg l^−1^ kanamycin and died, thus, eliminating regeneration of non-transgenic plants. Although the reason for such an escape in tree transformation is still unclear, there are some plausible explanations. Yevtushenko and Misra^18^ considered that non-transgenic cells could survive since nearby transformed cells caused to select incompletely. Nevertheless, very high antibiotic selection pressure is ineffective in this case because it simultaneously inhibits the transformed cell growth. Such an escape could be effectively removed by the second selection on a rooting medium with the antibiotic. Additionally, transgenic shoots that were induced from apical meristem tissues as transforming explants were frequently chimeric^35^. Confirming transgenic integration into each regenerated plant is an effective way to overcome this barrier for transgenes of trees.

In summary, we established a novel protocol for genetic transformation and regeneration of Nisqually-1. The protocol is so simple, rapid, efficient and reproducible that tree researchers easily introduce a variety of genes into this model tree and produce large-scale numbers of transgenic lines. Moreover, based on this protocol, we have developed highly-efficient target gene mutagenesis in this model tree using the CRISPR/Cas9 (clustered regularly interspaced short palindromic repeats/CRISPR-associated protein 9; unpublished data) system. In a nutshell, this basic tool for the first sequenced model tree removes a bottleneck to facilitate molecular and genetic studies on tree unique traits.

## Materials and Methods

### Plant materials

Shoots were cut from young trees of Nisqually-1 grown in the greenhouse (26/20 °C day/night, 16 h photoperiod and approximately 200 µmol photons m^−2^ s^−1^) and disinfected using 75% ethanol for 1 min and 5% sodium hypochlorite for 20 min. Then, the shoots were triple rinsed with sterile water and inserted in rooting medium (RM) that contained Lloyd & McCown Woody Plant Basal Medium w/Vitamins (WPM; PhytoTech Lab, L449), 25 g l^−1^ sucrose, 5.0 g l^−1^ agar and 0.1 mg l^−1^ indole-3-butyric acid (IBA) adjusted to pH 5.7. The resulting sterile plantlets were removed, apical and axillary buds were generated, which were later cut for rooting in RM. The obtained plantlets were used for subsequent experiments using the above described method.

### Optimization of shoot regeneration conditions

When individual plantlets showed 5 internodes and 6 leaves (Fig. 1a), internode stem segments and leaf petioles were excised into 0.8 to 1.0 cm in length as explants, and cultured on shoot regeneration medium (SRM) containing WPM, 25 g l^−1^ sucrose and 5.0 g l^−1^ agar (pH 5.7), which supplied various combinations of PGRs including 6-benzylaminopurine (6-BA), TDZ, IBA and NAA. Five groups of PGR combinations were included: (a) TDZ and NAA (0.01–0.2 and 0.01–0.2 mg l^−1^); (b) 6-BA and NAA (0.05–0.5 and 0.005–0.15 mg l^−1^); (c) 6-BA and IBA (0.04–0.5 and 0.02–0.1 mg l^−1^); d) 6-BA, NAA and TDZ (0.05–0.1, 0.025–0.1 and 0.0005–0.05 mg l^−1^); (e) 6-BA, IBA and TDZ (0.025–0.2, 0.02–0.1 and 0.0002–0.05 mg l^−1^). After ~25 days, the effect of each hormone treatment on shoot regeneration was observed. Based on the large-scale screens above, this group of combinations of 6-BA, IBA and TDZ emerged for further optimizing shoot regeneration. The effects of IBA and TDZ concentrations (0–0.04 and 0–0.0015 mg l^−1^) on shoot regeneration were evaluated on SRM in combination with 6-BA set to 0.03 mg l^−1^, and the number and length of shoots were calculated. Each hormone treatment contained 45 explants, and three replicates were performed. For shoot generation potential of different developmental internodes and leaf petioles, they were excised from each plantlet and cultivated on optimal SRM with 6-BA, IBA and TDZ. Ninety individual plantlets were taken as three replicates, and the number of shoots was calculated on average. Each 30 shoots with the length of 1–2 cm were cut and transferred to RM for rooting, and four replicates were performed. Tissue cultures were maintained in a room at 23–25 °C under a 16 h photoperiod and 50–70 µmol photons m^−2^ s^−1^.

### Kanamycin concentration for selection of transformants

For kanamycin concentration on shoot regeneration selection, the 2^nd^ and 3^rd^ internode stem segments were cut from 4-week-old plantlets and placed on optimal SRM that contained PGRs (6-BA, IBA and TDZ), cefotaxime (250 mg l^−1^) and different concentration of kanamycin (0, 10, 20, 30, 40 or 50 mg l^−1^). For rooting selection, the individual shoots were separated from explants and cultivated on RM that contained cefotaxime (250 mg l^−1^) and different concentration of kanamycin (0, 10, 20, 30, 40 or 50 mg l^−1^). After 28 days, shoot regeneration and induction of rooting were observed. All experiments were performed with three independent replicates, and each contained 21 stem segments or 18 individual shoots.

### Vector and preparation of *Agrobacterium tumefaciens* for transformation

*Agrobacterium tumefaciens* strain GV3101 was used for genetic transformation of Nisqually-1. The binary expression vector pBI_121_ (Supplementary Figure S3), which contains a β-glucuronidase (GUS) reporter gene drived by cauliflower mosaic virus (CaMV) 35S promoter, was transformed into strain GV3101 using the freeze-thaw method^36^. A single colony of *Agrobacteria* carrying pBI_121_ binary vector was inoculated in liquid Luria-Bertani medium (LB; 0.5% NaCl, 1% yeast extract and 1.6% tryptone) containing 50 mg l^−1^ rifampicin, 50 mg l^−1^ gentamicin and 50 mg l^−1^ kanamycin. The bacterial cultures were grown to 0.8~1.0 OD_600_ at 28 °C with 200 rpm. A 0.6 ml cultures were transferred to 30 ml fresh liquid LB with appropriate antibiotics. The cultures were incubated at 28 °C until an OD_600_ of 0.5~0.6 and were then, centrifugated immediately at 2200 *g* for 10 min before infection. The cell pellet was suspended in approximately 30 ml half-strength liquid WPM containing 2.5% sucrose adjusted to pH 5.2, generating the *A. tumefaciens* cell suspension with an OD_600_ of 0.4 for transformation.

### Evaluation of the factors affecting transformation frequency

Four factors were evaluated on transformation frequency: *Agrobacteria* suspension optical density (OD_600_ = 0.2, 0.4, 0.6, 0.8 or 1.0), *Agrobacteria* suspension infection duration (5, 10, 20 or 30 min), co-cultivation duration (0, 1, 2, 3 or 4 days) and acetosyringone (AS) concentration (0, 40, 80,160 or 320 µM) in *Agrobacteria* infective suspension and co-cultivation SRM. Stem segments of approximately 1 cm in length each were excised from the 2^nd^ to 3^rd^ internodes of individual plantlets with 5 internodes and 6 leaves. Stem segments were submerged in *Agrobacteria* suspension and slightly shaken periodically. Excess bacterial suspensions were removed from the explants after which they were placed on optimal SRM for co-cultivation. After co-cultivation, the explants were submerged in 250 mg l^−1^ cefotaxime water for 5 mins and washed three times with sterile water (each wash for 5 mins) to decontaminate *Agrobacteria*. The explants were then transferred into optimal SRM containing PGRs (6-BA, IBA and TDZ), kanamycin (30 mg l^−1^) and cefotaxime (250 mg l^−1^) for inducing kanamycin-resistant shoots. Each treatment contained 40 explants and three replicates were performed for each treatment. All kanamycin-resistant shoots were detected by GUS histochemical staining and transformation frequency was calculated as follows: the number of explants regenerating GUS-positive shoots/total number of cultured explants.

### Genomic DNA-PCR, Southern blot and RT-PCR analyses

Genomic DNA was extracted from the leaves of transgenic and control plants that were grown on soil for 8 weeks. Using these genomic DNAs as templates, the 625 bp DNA fragments of *GUS* gene was amplified using a pair of primers (5′-GGGCGAACAGTTCCTGATTAACC-3′ and 5′-CAGTACCTTCTCTGCCGTTTCCA-3′). Additionally, the *npt* III gene DNA fragment (243 bp) was amplified with pBI_121_ binary vector or transgenic plant genomic DNAs as templates and the primers were 5′-GATGTTGCTGTCTCCCAGGTCG-3′ and 5′-GCGGAGTGCATCAGGCTCTTTC-3′. For Southern blot analysis, each sample genomic DNA (20 μg) was digested with *Hind* III or *Eco* RI and consequent manipulations were carried out as described by Hai *et al*.^23^ For *GUS* gene expression in non-transformed and transgenic plants, RNA extraction and cDNAs synthesis were performed as described by Hai *et al*.^23^. The *GUS* gene was detected at transcriptional level by reverse transcriptional PCR and its primers were the same as mentioned above. *Populus Actin2* was selected as reference gene to quantify each cDNA template and the primers were 5′-TCCATCACCAGAATCCAGCACA-3′ and 5′-AACATGGGATTGTTAGCAACTGG-3′.

### GUS staining assay

The 2-month-old control and transgenic young trees in the greenhouse were used for GUS staining analysis. Various tissues of each sample were stained using the method as described by Jefferson *et al*.^37^. After GUS staining, 70% (v/v) ethanol was used for removing the chlorophyll.

### Statistical analysis

The SPSS 18.0 (Chicago, IL, USA) was used for all the data analyses. The value *p* < 0.05 was considered to be statistically significant.

## Electronic supplementary material

Simple, rapid and efficient transformation of genotype Nisqually-1: a basic tool for the first sequenced model tree
