# Supplementary material for: Simple, rapid and efficient transformation of genotype Nisqually-1: a basic tool for the first sequenced model tree
Source: Sci Rep. 2017 Jun 1;7:2638. doi: 10.1038/s41598-017-02651-x (PMC5453977; doi:10.1038/s41598-017-02651-x)
Supplement: Supplementary file 1 — Simple, rapid and efficient transformation of genotype Nisqually-1: a basic tool for the first sequenced model tree [file 41598_2017_2651_MOESM1_ESM.pdf]

Simple, rapid and efficient transformation of genotype Nisqually-1: a basic tool for the first sequenced model tree

Shujuan Li[1#], Cheng Zhen[1#], Wenjing Xu[1], Chong Wang[1], Yuxiang Cheng[1*]

[1] State Key Laboratory of Tree Genetics and Breeding, Northeast Forestry University, 26 Hexing Road, Harbin 150040, China

*Corresponding author: Yuxiang Cheng; chengyuxiang@nefu.edu.cn

[#] These authors contributed equally to this work.

Supplementary data

Figure S1. Effect of kanamycin on shoot regeneration from stem segments of Nisqually-1. The 2th and 3th internode stem segments were cut from 25-day-old plantlets and placed on optimal SRM with PGRs (6-BA, IBA and TDZ) and 250 mg l^{-1} cefotaxime supplemented with 0, 10, 20, 30, 40 and 50 mg l^{-1} kanamycin (a-f). After 4 weeks, induction of shoot regeneration was observed. Three replicates were performed, and each contained 21 stem segments.

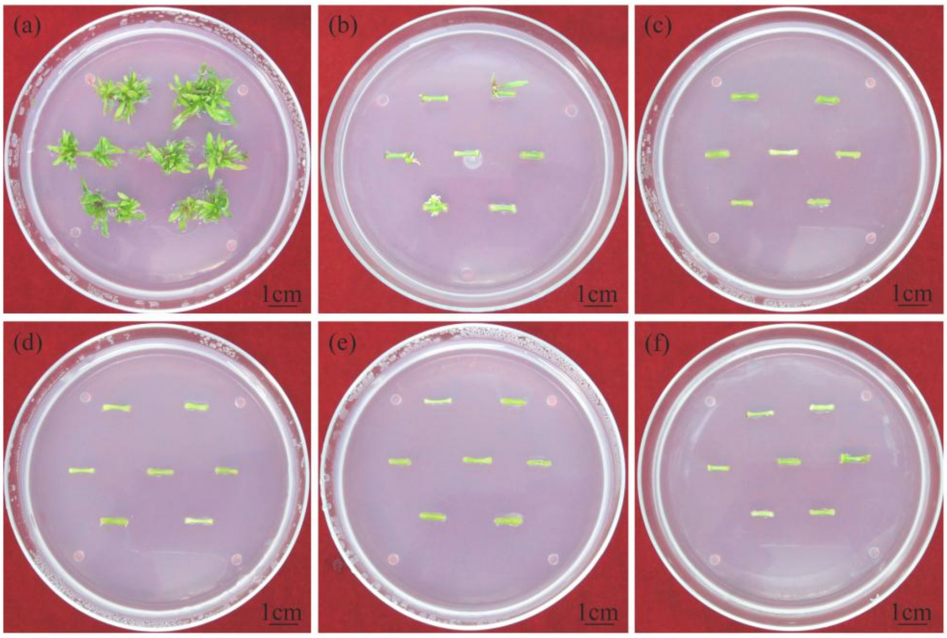

Figure S2. Effect of kanamycin on rooting of shoots on RM. The 1-2 cm individual shoot was separated from explants and cultured on RM with 250 mg l^{-1} cefotaxime supplemented with 0, 10, 20, 30, 40 and 50 mg l^{-1} kanamycin (a-f). After 4 weeks, root induction was observed. Three independent replicates were performed and each kanamycin treatment contained 18 individual shoots.

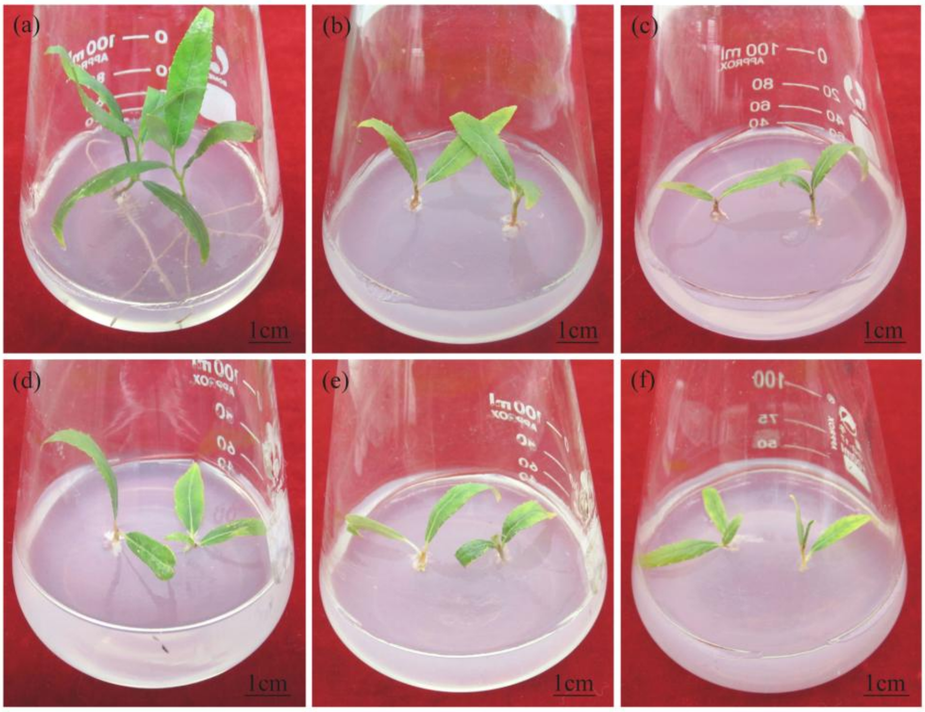

Figure S3. pBI121 plant expression vector for the *Agrobacterium*-mediated transformation of Nisqually-1. Two primer sets (P1 and P2) for molecular analyses of CaMV 35S::GUS transgenic plants were shown in the vector.

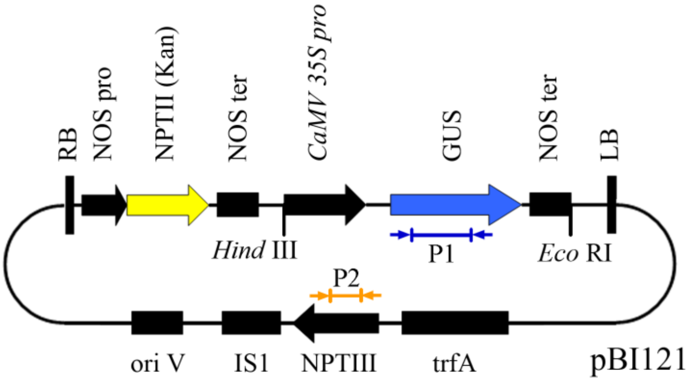

Table S1. Screening of plant growth regulator (PGR) combinations for induction of multiple shoots from stem segments of Nisqually-1

Combinations of PGRs (mg l^{-1})	Induction of shoots
TDZ (0.01-0.2) + NAA (0.01-0.2)	blackish green callus, no shoot
6-BA (0.05-0.5) + NAA (0.005-0.15)	seldom
6-BA (0.04-0.5) + IBA (0.02-0.1)	seldom
6-BA (0.05-0.1) + NAA (0.025-0.1) + TDZ (0.0005-0.05)	few shoots
6-BA (0.025-0.2) + IBA (0.02-0.1) + TDZ (0.0002-0.05)	cluster shoots

Table S2. Regenerating potential of different internode stem segment explants of Nisqually-1.

Internodes	Regeneration frequency (%)	Shoot number per explant	Shoot length (cm)
1st internode stem segment	88.67±1.16b	7.93±1.55c	1.03±0.12ab
2nd internode stem segment	100.0±0.0a	15.20±1.15a	1.07±0.10ab
3rd internode stem segment	100.0±0.0a	16.57±0.51a	1.15±0.02a
4th internode stem segment	100.0±0.0a	11.87±0.51b	0.98±0.05c

Four internodal stem segments (from top to bottom) of each individual 25-day-old plantlet were cut and placed on optimal SRM with 6-BA, IBA and TDZ for shoot regeneration. Ninety individual plantlets were performed for three replicates and each contained ten explants. Values are means ± standard error. Values with the different letters indicate statistical significance at $P < 0.05$ level (Duncan's multiple range test).

Table S3. Regenerating potential of different leaf petiole explants of Nisqually-1.

Leaf petioles	Regeneration frequency (%)	Shoots number per explant	Shoot length (cm)
Petiole of 1st leaf	78.87±5.10c	5.11±0.26d	0.90±0.033e
Petiole of 2nd leaf	94.43±5.10ab	7.05±0.25c	0.99±0.037d
Petiole of 3rd leaf	100.00±0.00a	12.11±0.26a	1.21±0.013a
Petiole of 4th leaf	100.00±0.00a	12.5±0.17a	1.19±0.018ab
Petiole of 5th leaf	100.00±0.00a	11.72±0.25a	1.20±0.020ab
Petiole of 6th leaf	100.00±0.00a	9.22±1.45b	1.15±0.030bc

Six leaf petioles (from top to bottom) of each 25-day-old plantlet were cut and placed on optimal SRM with 6-BA, IBA and TDZ for shoot regeneration. Ninety individual plantlets were performed for three replicates, and each contained ten explants. Values are means ± standard error. Values with the different letters indicate statistical significance at $P < 0.05$ level (Duncan's multiple range test).
